# Polysaccharide Utilization and Adhesion Enable the Genome-Streamlined *Opacimonas immobilis* to Adapt to the Diatom Phycosphere

**DOI:** 10.3390/microorganisms14010139

**Published:** 2026-01-08

**Authors:** Xiaoyu Yang, Xuanru Lin, Jianmin Xie, Runlin Cai, Guanjing Cai, Hui Wang

**Affiliations:** 1 Guangdong Provincial Key Laboratory of Marine Biotechnology, Shantou University, Shantou 515063, China; 19xyyang@stu.edu.cn (X.Y.);; 2 Biology Department and Institute of Marine Sciences, College of Science, Shantou University, Shantou 515063, China; 3 Guangdong Engineering Technology Research Center of Offshore Environmental Pollution Control and Shantou Key Laboratory of Marine Microbial Resources and Interactions with Environment, Shantou University, Shantou 515063, China

**Keywords:** phycosphere, genome reduction, diatom, motility, polysaccharides utilization, adhesion

## Abstract

Heterotrophic bacteria and microalgae are key regulators of marine biogeochemical cycles. The phycosphere, a nutrient-rich microenvironment surrounding microalgae, serves as a crucial interface for bacterial–algal interactions. Our previous work identified *Opacimonas immobilis* LMIT016^T^, a phycosphere isolate from the diatom *Actinocyclus curvatulus* that possesses the smallest genome within the *Alteromonadaceae* family. However, its adaptation mechanisms to the phycosphere remain unclear, particularly given its extensive genome streamlining, a process involving the selective loss of non-essential and energetically costly genes to enhance fitness in nutrient-specific niches. Here, the co-cultivation experiments demonstrated significant mutual growth promotion between LMIT016^T^ and its host microalgae, with the bacterium forming dense attachments on diatom surfaces. Genomic analysis revealed that in addition to loss of motility-related genes, the strain exhibits a substantial reduction in c-di-GMP signaling components, including both synthases and receptors. Conversely, LMIT016^T^ harbors numerous genes essential for extracellular polysaccharide (EPS) biosynthesis and adhesion, supporting long-term attachment and biofilm formation. Other retained genes encode pathways for nutrient acquisition, stress response, and phosphate and nitrogen metabolism, reflecting its adaptations to the nutrient-rich phycosphere. Furthermore, the genome of LMIT016^T^ encodes two polysaccharide utilization loci (PULs) targeting laminarin and α-1,4-glucans, whose functions were experimentally validated by the transcriptional induction of the corresponding carbohydrate-active enzyme genes. These findings indicate that this strain counterbalances genome reduction by enhancing its attachment capabilities and metabolic specialization on algal polysaccharides, potentially facilitating stable association with diatom cells. Our results suggest that genome streamlining may represent an alternative ecological strategy in the phycosphere, highlighting a potential evolutionary trade-off between metabolic efficiency and niche specialization.

## 1. Introduction

The ecological relationship between phytoplankton and bacteria represents one of the most influential processes in the marine environment [1]. As the basis of the marine food web, phytoplankton contribute nearly half of the total primary production on Earth [2] and release part of their photosynthetic products, forming a nutrient-rich phycosphere that attracts bacterial colonization [3,4]. Within this microenvironment, phytoplankton-derived organic compounds such as amino acids and polysaccharides serve as nutrients for diverse associated bacteria, including members of the Roseobacter clade and Flavobacteria [5]. In turn, bacteria can stimulate algal growth by supplying essential substances such as nutrients, vitamins, and signaling molecules [6,7,8,9,10]. The interaction between phytoplankton and bacteria modifies the surrounding chemical environment and the physiology of both partners, ultimately influencing the global ecosystem [11,12].

Bacteria within the family *Alteromonadaceae* (*Gammaproteobacteria*) are acknowledged as one of the most prevalent and abundant groups in the phycosphere [13,14]. These microorganisms play pivotal roles in marine carbon and nitrogen cycling owing to their exceptional capacity to metabolize diverse organic substrates [15,16]. This metabolic versatility endows *Alteromonadaceae* with typical copiotroph characteristics. They exhibit low abundance in oligotrophic conditions (e.g., early algal blooms), but undergo rapid and opportunistic proliferation in response to transient nutrient pulses during late-stage blooms or in eutrophic zones [17,18]. This copiotrophic strategy is primarily facilitated by the large genomes of *Alteromonadaceae* members, which encode extensive enzymatic networks. These networks enable the utilization of diverse phytoplankton-derived carbon sources, including complex polysaccharides such as laminarin, alginate, agarose, and pectin [19,20,21]. These metabolic adaptations are further supported by genome plasticity, with flexible regions enriched with gene clusters that enhance stress resistance and nutrient utilization efficiency [22]. Additionally, traits such as chemotaxis and motility enable bacterial strains respond rapidly and move toward nutrient-rich environments [23].

In addition to copiotrophic bacteria with large and flexible genomes, genome streamlining has been widely documented as an ecologically successful strategy among marine bacteria. Genome streamlining involves reducing genome size and losing non-essential functions to optimize core metabolic processes. This strategy occurs in both free-living and symbiotic bacteria, often driven by long-term adaptation to specific ecological niches [24,25]. In our previous study [26], we found that a genome-reduced strain of the family *Alteromonadaceae*, *Opacimonas immobilis* LMIT016^T^, successfully adapted to the phycosphere. Strain LMIT016^T^ possesses the smallest genome (2.53 Mb) within the family, approximately 43% smaller than the average genome size of other bacteria in the family. Consistently, both its total gene count and coding gene numbers are markedly lower than those of all reference type strains. Genome annotation revealed the absence of most genes involved in flagellar assembly and chemotaxis, despite these genes being typically conserved among other family members. Despite extensive genomic reduction, this strain has retained functional genes potentially associated with niche adaptation, including a diverse repertoire of carbohydrate-active enzymes and complete Type IV pili and Type II secretion systems [26]. However, the mechanism by which this genome-streamlined strain adapts to the diatom phycosphere remains poorly understood.

To elucidate how genome reduction and gene retention facilitate niche adaptation of strain LMIT016^T^, we compared its growth in a diatom–bacterial co-culture system with that of reference *Alteromonadaceae* strains, thereby identifying key traits associated with phycosphere colonization. Subsequently, we analyzed gene categories implicated in marine bacterial–host interactions to identify the key genomic features that facilitate the colonization of strain LMIT016^T^ in the phycosphere, and we further validated these features experimentally. Our findings could uncover an adaptive genome reduction strategy that may fundamentally influence microalgal–bacterial interactions and their co-evolution.

## 2. Materials and Methods

### 2.1. Bacterial and Microalgal Strains

Strain LMIT016^T^ was isolated from the liquid culture of the diatom *Actinocyclus curvatulus* CNS00114, a species widely distributed in global oceans and an important member of marine phytoplankton communities, contributing to carbon fixation and primary production in marine ecosystems [27]. The bacterial strain has been preserved in our laboratory as a glycerol stock (20% (*v*/*v*) glycerol and stored at −80 °C) [26]. Two type strains in the family of *Alteromonadaceae*, *Alteromonas lipolytica* JW12^T^ and *Alteromonas halophila* KCTC 22164^T^, purchased from the Korean Collection for Type Cultures, were used as reference strains to facilitate comparative analyses of the genomic and metabolic potential of strain LMIT016^T^. The axenic diatom strain *Phaeodactylum tricornutum* CCAP 1055 provided by Dr. Xiaojuan Liu from Shantou University was cultured in sterile f/2 medium [28] with a light/dark cycle of 12:12 at 20 °C and light intensity of 140 ± 20 µmol photons m^−2^ s^−1^ incubating conditions.

### 2.2. Monitoring of Diatom–Bacterial Co-Cultivation Systems

Strain LMIT016^T^ and two reference strains were separately co-cultured with the axenic diatom strain CCAP 1055. The diatom strain was pre-cultured to mid-exponential phase with a cell density of 1 × 10^6^ cells ml^−1^ in f/2 medium. Bacterial strains were pre-cultured in 2216E marine broth to mid-exponential phase with a cell density of 2 × 10^9^ cells ml^−1^. Liquid cultures of bacteria were centrifuged at 9500 g for 10 min, and the resulting pellets were washed and resuspended with sterilized f/2 medium twice. Bacterial cells were then inoculated into the pre-cultured axenic diatom cultures with an initial density of 5 × 10^7^ cells ml^−1^, corresponding to an initial diatom-to-bacterium inoculation ratio of approximately 1:50. Co-cultures were incubated under a light/dark cycle of 12: 12 at 20 °C and light intensity of 140 ± 20 µmol photons m^−2^ s^−1^ for four days. The growth of the diatom and bacteria in the co-cultures was quantified based on cell density (cells ml^−1^) using a flow cytometer (BD Accuri^TM^ C6 Plus, BD Biosciences, Franklin Lakes, NJ, USA). All experiments were conducted with three independent biological replicates.

Transparent exopolymer particles (TEP) associated with the attachment between the diatom cells and bacteria were detected using an inverted microscope. Briefly, aliquots (20 µL) of the above diatom–bacterial co-cultures were collected after one day of incubation, placed on microscope slides and stained with Alcian Blue (Macklin, Shanghai, China) for 10 min [29]. Excess Alcian Blue was washed away with PBS (phosphate-buffered saline) buffer. Subsequently, SYBR Green I (Macklin, Shanghai, China) was added to stain the nucleic acid in the dark for 10 min, followed by PBS washing to remove residual dye. The slides were observed by an inverted microscope (Axio Observer, ZEN, Carl Zeiss, Oberkochen, Germany) with the bright field and fluorescence, respectively. Images captured at bright-field and fluorescence were merged using ZEN Blue software v. 3.10 (https://www.zeiss.com.cn/microscopy/home.html, accessed 10 September 2024). To quantify the bacterial attachment, the adhesion density was calculated as the number of bacterial cells per unit surface area of diatoms (cells/µm^2^). ImageJ software v. 1.54g was used to manually trace the diatom boundaries for area measurement and to count individual attached bacteria across multiple fields of view from three biological replicates. All analyses were performed using GraphPad Prism software v. 10.2.3 [30].

### 2.3. Comparative Genomic Analysis of Interaction-Related Genes

The complete genome of strain LMIT016^T^ was sequenced previously and deposited in the NCBI GenBank database under the accession number GCA_048282545.1 [26]. Two *Alteromonadaceae* reference strains, *A. lipolytica* JW12^T^ and *A. halophila* KCTC 22164^T^, were included for comparative genomic analyses, with genome sequences retrieved from the NCBI GenBank database (https://www.ncbi.nlm.nih.gov/genbank/, accessed on 10 January 2024) under accession numbers GCA_001758465.1 and GCA_014651815.1, respectively. In addition, genomes of reported biofilm-forming marine bacteria, including *Stappia indica* PHM037 (GCA_009789575.1), *Pseudoalteromonas tunicata* D2 (GCA_003568825.1), and *Vibrio vulnificus* MO6-24/O (GCA_000186585.1), were also downloaded from the NCBI GenBank database and used for capsular polysaccharide (CPS) biosynthesis gene cluster comparison.

Prokka v1.14.0 (https://github.com/tseemann/prokka, accessed on 15 January 2024) and eggnog-mapper (v2.1.11, https://github.com/eggnogdb/eggnog-mapper/wiki, accessed on 15 January 2024) were applied to predict protein-coding genes via the KEGG database (https://www.kegg.jp/blastkoala/, accessed on 15 January 2024), and to annotate genome annotation, respectively. Gene categories mediating marine bacterial–host interactions were analyzed [31], including quorum sensing (QS), capsular systems, lipopolysaccharide synthesis, carbohydrate-active enzymes (CAZymes), TonB-dependent transporters (SusCD), biosynthetic gene clusters (BGCs), antibiotic resistance (Anti_res), and phosphorus and nitrogen metabolism (P/N metabolism). Specifically, the capsular systems were annotated via an online website with default parameters (https://research.pasteur.fr/en/tool/capsulefinder/, accessed on 5 April 2025). PULs (polysaccharides utilizing locus) were identified using the dbCAN2 database (https://bcb.unl.edu/dbCAN2/, accessed on 15 January 2024) and eggNOG database (https://github.com/eggnogdb/eggnog-mapper/wiki, accessed on 15 January 2024) following the delimiting criterion by Mann et al. [32]. Gene clusters associated with candidate genes were defined as PULs if containing at least one transporter protein and at least two CAZymes. BGCs were annotated in online website (https://antismash.secondarymetabolites.org/, accessed on 15 January 2024) with default parameters.

### 2.4. Detection of Polysaccharides Utilizing Activity

The utilization of algae-derived polysaccharides was analyzed by inoculating and incubating strain LMIT016^T^ in seawater minimal medium (SWM) [33] supplemented with 0.1% (g/v) different alga-derived polysaccharides (starch, laminarin, agarose, pectin, sodium alginate, xyloglucan) as the sole carbon source. Strain was pre-cultured in 2216E marine broth at 25 °C and 150 rpm until the OD_600_ value reached 0.6–0.7. Bacterial cells were collected by centrifuging at 9500 g. After being washed and resuspended twice with SWM, cells were inoculated into SWM containing 0.1% of different polysaccharides (starch, laminarin, agarose, pectin, sodium alginate, xyloglucan) and incubated at 25 °C, 150 rpm. The SWM supplemented with 0.1% polysaccharide, but without the addition of bacteria, was used as a negative control. All the experiments were performed in triplicate.

Bacterial growth was determined by photometry (OD_600_). The Fluorophore-assisted carbohydrate electrophoresis (FACE) was applied to analyze the degradation of laminarin. Briefly, after being cultured in SWM supplemented with polysaccharides, the supernatant of the strain LMIT016^T^ was collected every 24 h by centrifugation (12,000 rpm, 2 min). The reducing end of residual carbohydrates in the supernatants was labeled with 8-aminonaphthalene-1,3,6-trisulfonic acid (ANTS). Labeled sugars were further separated in polyacrylamide gel as described by Robb et al. [34]. Gels were then visualized and imaged using a standard UV light imager (ChiTang WFH_203C, Shanghai Jingke, Shanghai, China).

### 2.5. qRT-PCR Detection of Glycoside Hydrolase-Encoding Genes

Quantitative real-time polymerase chain reaction (qRT-PCR) was applied to quantify the expression of laminarin-hydrolyzing genes, specifically glycoside hydrolase (GH) family genes GH1, GH3, and GH16. Specific primers targeting these glycoside hydrolase-encoding genes were designed using the NCBI online tool (https://www.ncbi.nlm.nih.gov/tools/primer-blast/, accessed on 15 August 2024), and the primer sequences are listed in Supplementary Table S1. Bacterial cells were harvested from the laminarin utilization experiment described above at 12 h and 24 h of incubation. Cells grown in SWM without laminarin and harvested at the corresponding time points were used as controls. Cultures were centrifuged at 5000 g for 2 min at 25 °C, and the supernatant was discarded prior to RNA extraction. Total RNA was then extracted using the E.Z.N.A.^®^ Bacterial RNA Kit (R6950-02, Omega, Biel/Bienne, Switzerland). Reverse transcription and real-time PCR were performed by using the StarScript II First-strand cDNA Synthesis Kit (A214-02, GenStar, Beijing, China) and 2 × RealStar Green Fast Mixture (A301-10, GenStar, Beijing, China). All samples were analyzed in technical triplicate. The PCR conditions were as follows: initial denaturation at 95 °C for 2 min, then 40 cycles of denaturation at 95 °C for 15 s, annealing at 60 °C for 30 s and extension at 60 °C for 6 s. Relative transcript abundances were calculated using the 2^−ΔΔCt^ method, with normalization to the 16S rRNA gene [35,36]. Transcript signals for each treatment were compared to those of the control group. Statistical significance between treatments and controls was assessed using Student’s *t*-test.

## 3. Results

### 3.1. Strain LMIT016^T^ Exhibited the Steadiest Growth While Benefiting the Diatom

**Figure 1:**
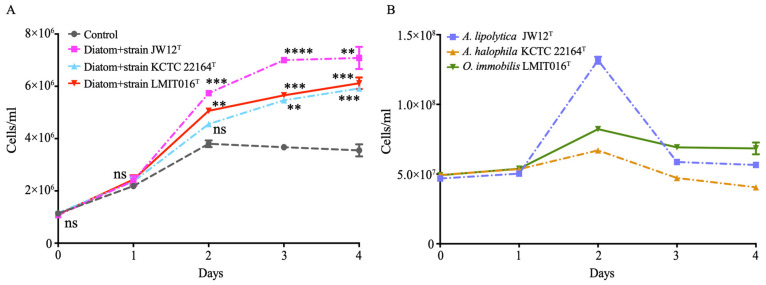
Co-culture of bacteria with diatoms shows the mutual growth of diatom cells (**A**) and bacteria (**B**). The diatom and bacterial strains were co-inoculated at an initial ratio of approximately 1:50 in f/2 medium. Co-cultures were incubated at 20 °C under a 12:12 h light/dark cycle (140 ± 20 µmol photons m^−2^ s^−1^) for four days. Data are presented as mean ± SD from three biological replicates. Statistical significance of diatom growth promotion in panel A by strain LMIT016^T^ relative to the axenic diatom control was assessed using Student’s *t*-test; ns, not significant; **, *p* < 0.01; ***, *p* < 0.001; ****, *p* < 0.0001.

To investigate the interaction between strain LMIT016^T^ and its diatom host, we monitored their co-culture dynamics and compared them with those of two reference strains (JW12^T^ and KCTC 22164^T^). Notably, co-cultivation with strain LMIT016^T^ resulted in a steady and continuous increase in diatom abundance, reaching 6.2 × 10^6^ cells mL^−1^ on day 4 (Figure 1A). This final abundance was significantly higher than that of the axenic control, corresponding to a 1.72-fold increase, and was comparable to the growth-promoting effect of strain JW12^T^ (2.00-fold increase), while still slightly exceeding the enhancement observed with strain KCTC 22164^T^ (1.67-fold increase, Figure 1A).

The three bacterial strains exhibited distinct growth patterns when co-cultured with the diatom (Figure 1B). Strain LMIT016^T^ demonstrated steady growth, increasing from 5.0 × 10^7^ cells mL^−1^ to 8.0 × 10^7^ cells mL^−1^ on day 2, and stabilizing moderately thereafter until day 4. In contrast, the reference strain JW12^T^ showed a rapid initial increase at day 2 but subsequently declined by more than 50%, ultimately falling to a level lower than the final abundance of strain LMIT016^T^ by day 4. Strain KCTC 22164^T^ demonstrated the lowest growth, reaching only 6.0 × 10^7^ cells mL^−1^ before gradually decreasing. Collectively, these patterns indicated that strain LMIT016^T^ maintained more stable populations while consistently supporting diatom growth, indicative of a mutually beneficial association.

### 3.2. LMIT016^T^ Cells Adhered and Formed Dense Aggregates on the Diatom Surface

**Figure 2:**
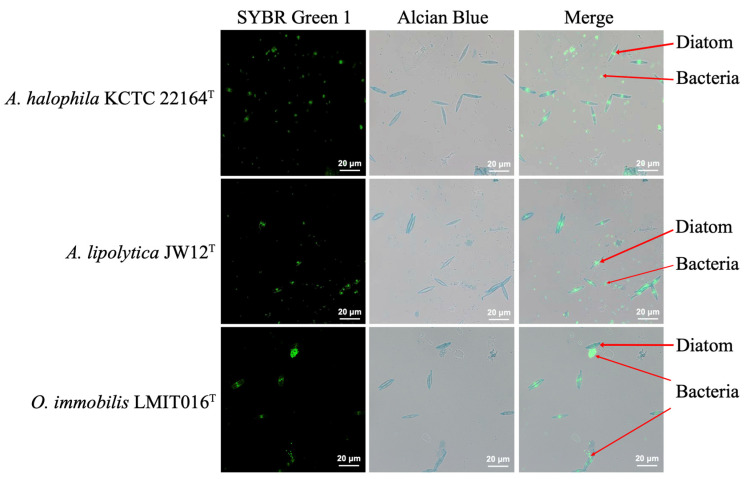
Fluorescence micrograph showing diatom–bacterial adhesion. Diatom cell walls were stained with Alcian Blue (blue). The DNA of diatoms and bacteria was stained with SYBR Green I (green). Composite images visualize attachment interfaces. Scale bars: 20 µm (100× objective).

To further investigate the physical interactions underlying the different growth responses of LMIT016^T^ and the reference strains, we assessed the morphology of microalgal-bacterial co-culture using combined bright-field and fluorescence microscopy. The results demonstrated that a substantial number of LMIT016^T^ cells adhered to the diatom surface, forming dense aggregates. In contrast, cells of strains JW12^T^ and KCTC 22164^T^ remained predominantly free-living and dispersed away from the diatoms (Figure 2 and Figure S1). Quantitative analysis confirmed a significant difference in attachment density among the three strains (*p* = 0.02, Kruskal–Wallis test, Figure S2). Specifically, strain LMIT016^T^ exhibited a higher adhesion density (0.4786 ± 1.4883 cells/µm^2^) than those of JW12^T^ (0.0385 ± 0.0487 cells/µm^2^) and KCTC 22164^T^ (0.0059 ± 0.0116 cells/µm^2^). These observations suggested that LMIT016^T^ adopts an attachment-oriented lifestyle, which may underlie its distinct growth behavior in the co-culture.

### 3.3. Strain LMIT016^T^ Exhibited Genomic Features Supporting Stable Surface Association with the Diatom

**Figure 3:**
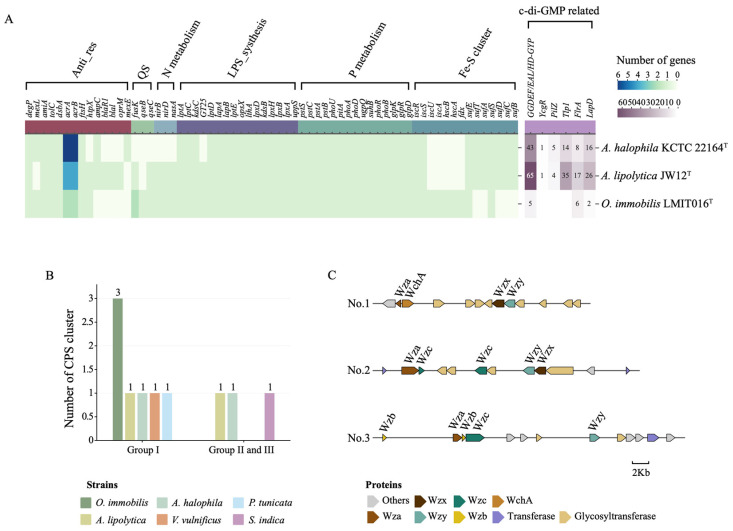
Functional annotation of genes involved in marine bacterial interactions. (**A**) Gene sets mediating marine bacterial–host and bacterial–bacterial interactions annotated in genomes of strains LMIT016^T^, KCTC 22164^T^ and JW12^T^. Color intensity corresponds to the number of genes per strain. (**B**) Number of different capsular systems identified in each strain. (**C**) Annotation of three capsule single loci within the genome of strain LMIT016^T^.

To elucidate the genetic basis underlying the attachment-oriented lifestyle of strain LMIT016^T^ in the diatom phycosphere, we performed a detailed genomic analysis. Notably, beyond the previously reported loss of flagellar and chemotaxis systems [26], LMIT016^T^ exhibited a pronounced reduction in the genomic potential for bis-(3′-5′)-cyclic dimeric guanosine monophosphate (c-di-GMP) signaling (Figure 3A). Specifically, LMIT016^T^ encodes only five GGDEF-, EAL-, and HD-GYP-domain–containing proteins, compared with 65 and 43 in reference strains JW12^T^ and KCTC22164^T^, respectively. Furthermore, numerous c-di-GMP effectors, including PilZ-domain, YcgR, LapD and Tlp1 receptors, were absent or markedly reduced in its genome. Given the established role of c-di-GMP in regulating transitions between motile and biofilm-associated states in bacteria [37,38], these genomic features suggest a substantially streamlined c-di-GMP regulatory network in LMIT016^T^, potentially constraining its capacity for dynamic lifestyle switching.

In addition, LMIT016^T^ possessed a complete genetic repertoire for extracellular polysaccharide (EPS) and lipopolysaccharide (LPS) biosynthesis (Figure 3A). Core lipid A biosynthesis genes (*lpxA, lpxB, lpxD,* and *lpxH; kdsB* and *kdsC*), LPS export components (*lptA–lptE*), glycosyltransferases including GT25, and modification enzymes encoded by *opsX* and *lthA* were all present, supporting robust outer-membrane structuring and remodeling [39]. It also encoded the large adhesion proteins LapA and LapB, which have been implicated in long-term surface attachment and biofilm stabilization [40]. Notably, although strain LMIT016^T^ lacked Group II capsular polysaccharide (CPS) loci, it harbored three complete Group I CPS loci, exceeding the single Group I locus detected in each reference strain and the reported biofilm-forming marine bacterial strains (Figure 3B). Each CPS cluster contained the Wza/Wzb/Wzc export system, Wzx flippase, Wzy polymerase, and diverse glycosyltransferases and epimerases, forming three syntenic operons (Figure 3C). The coexistence of multiple CPS loci suggests an expanded genetic repertoire for capsular polysaccharide production and structural diversity [41], which may contribute to cell-surface properties associated with attachment-related phenotypes observed in strain LMIT016^T^.

Besides the aforementioned features associated with surface attachment, LMIT016^T^ retained multiple genes associated with antibiotic resistance and envelope stress responses (Figure 3A), including *degP*, *mexL*, *amiA*, *tolC*, *dsbA*, *acrA*, *acrB*, *ftsH*, and *htpX*, which encode proteases, efflux components, and envelope quality-control factors contributing to antimicrobial tolerance [42]. Genes involved in quorum sensing (*qseB* and *qseC*) were also present, suggesting preserved basic cell-to-cell communication [43]. Collectively, these genetic traits likely enable LMIT016^T^ to maintain stable associations with diatoms in the complex and competitive marine environment.

### 3.4. Strain LMIT016^T^ Encodes Genes for Stable Energy Conversion and Nutrient Acquisition

To see why strain LMIT016^T^ exhibited the steadiest growth in the diatom phycosphere, we focused on genes associated with energy conversion and nutrient acquisition. Interestingly, the organization of Fe–S cluster biogenesis pathways diverged among the three genomes. LMIT016^T^ retained a complete *isc* system (*iscR, iscS, iscU, iscA, hscB, hscA, fdx,* and *iscX*) but lacked the *suf* system (*sufA-sufE*). In contrast, the two reference strains possessed the *suf* system but lacked a complete *isc* system (Figure 3A). The isc system typically serves as the primary housekeeping route for Fe–S assembly, whereas *suf* is induced under oxidative or iron-limited conditions [44]. The absence of the stress-inducible *suf* system suggests that LMIT016^T^ primarily relies on the basal *isc* machinery, consistent with adaptation to a relatively stable microenvironment.

For nitrogen metabolism, LMIT016^T^ encodes *nirB, nirD,* and *nasA*, indicating its capacity for assimilatory nitrite and nitrate reduction (Figure 3A). Regarding phosphorus acquisition, LMIT016^T^ maintained a complete phosphate uptake system (*pstS–pstC–pstA–pstB and phoU*), the full *phoB-phoR* regulatory module, and multiple phosphatases and transporters (*phoA*, *phoD*, *pitA*, *ugpQ*, *suhB*), reflecting strong adaptation to phosphate-limited conditions typical of the phycosphere [45] (Figure 3A). Additionally, conserved genes for glycerol-3-phosphate utilization (*glpKRD*) suggest metabolic flexibility for using algal-derived organics [46].

Since diatoms typically accumulate polysaccharides during the late exponential and stationary growth phases, which coincide with the period when LMIT016^T^ displays a population advantage over other strains, we further evaluated the polysaccharide utilization potential of LMIT016^T^. Consistently, two polysaccharide utilization loci (PULs) were identified in the genome of LMIT016^T^ (Table 1). Based on functional annotation of key CAZymes, PUL1 was predicted to target laminarin, a major algal β-1,3-glucan, whereas PUL2 was predicted to target α-1,4-glucans. Specifically, the laminarin PUL1 encoded genes for three key glycoside hydrolases: GH1 (β-glucosidases), GH3 (exo-β-1,3-glucanases) and GH16 (endo-β-1,3-glucanases). GH16 hydrolyzes the β-1,3 backbone into glucose and oligosaccharides, which were subsequently hydrolyzed into glucose monomers by GH3 and GH1 [47,48]. Beyond these conserved CAZymes, PUL1 also encoded transporters (TonB-dependent receptors and an MFS transporter), genes involved in monomer uptake and metabolism (e.g., *gnd*, *pfkB*, *glpK*, *glpD*), and the transcriptional regulator *glpR*. Collectively, these components constitute a complete laminarin utilization module. The α-1,4-glucan PUL2 encoded two α-glucan–degrading enzymes possessing a CBM48 carbohydrate-binding module: GH13 (α-amylase) and GH77 (α-1,4-glucanotransferase). Additionally, PUL2 contained genes encoding enzymes for monosaccharide metabolism (e.g., *gapA*, *yeaD*, *eda*, *glk*, *edd*, *pgl* and *zwf*), and the transcriptional regulator *hexR*, forming a dedicated pathway for the utilization of released sugars (Table 1). The presence of these two specialized PULs suggests that strain LMIT016^T^ possesses a broad capacity to utilize β-1,3-glucans (specifically laminarin) and α-1,4-linked glucans, potentially enabling it to thrive on polysaccharides produced by its diatom host.

### 3.5. Strain LMIT016^T^ Efficiently Utilize Laminarin and α-1,4-Glucan via Two PULs

**Figure 4:**
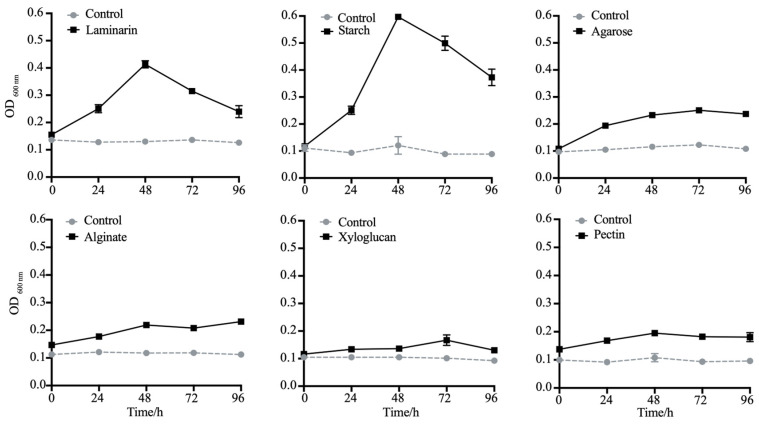
Growth of strain LMIT016^T^ on selected polysaccharides as the sole carbon source. Bacterial growth was measured by optical density(OD_600_) in modified basal medium supplemented with 0.1% (g/v) of each polysaccharide. Tested substrates include laminarin, starch, agarose, pectin, alginate, and xyloglucan. Solid lines represent bacterial growth on each polysaccharide; dotted lines represent the control (growth in carbon-free basal medium). Data are expressed as mean ± SD of three technical replicates.

**Figure 5:**
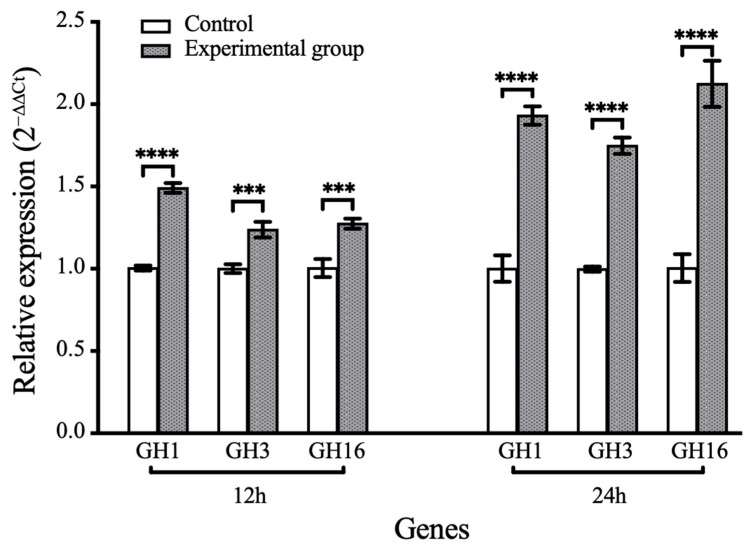
Relative expression levels of target genes during the laminarin degradation. Bars represent mean relative expression levels (2^−ΔΔCt^) ± SD (*n* = 3 biological replicates). Statistical differences between groups were assessed using Student’s *t*-test. Significance levels: **** *p* < 0.0001, *** *p* < 0.001.

To validate polysaccharides utilization capability, LMIT016^T^ was cultured using various algal-derived polysaccharides as sole carbon sources (Figure 4). The strain exhibited significant growth on laminarin and starch (reaching OD_600_ values of 0.6 and 0.4 at 48 h, respectively), but failed to grow on agarose, pectin, sodium alginate and xyloglucan. Given laminarin’s role as a major diatom-secreted polysaccharide [49], its utilization was further analyzed using FACE (Figure S3). Multiple bands visible at the first 24 h weakened considerably by 48 h and 72 h, coinciding with the appearance of bands corresponding to oligomers after 96 h, indicating progressive enzymatic degradation. Concurrently, qRT-PCR analysis demonstrated a significant induction of laminarin-degrading genes in strain LMIT016^T^ upon laminarin supplementation. Compared to controls, the relative expression levels of genes encoding GH1, GH3 and GH16 increased by 1.49-, 1.23-, and 1.27-fold at the early incubation stage, respectively. After 24 h of exposure to laminarin, their expression further increased to 1.93-, 1.75-, and 2.12-fold, respectively (Figure 5). All differences were statistically significant (*p* < 0.01). Collectively, these genomic and physiological findings demonstrate that LMIT016^T^ efficiently attaches to diatom cells and efficiently utilizes diatom-derived laminarin as a key nutrient source.

## 4. Discussion

The interactions between microalgae (particularly diatoms) and bacteria are now recognized as widespread and ecologically important processes in aquatic ecosystems, involving reciprocal exchanges of nutrients, metabolites, and signaling molecules [1,12,50]. *Alteromonadaceae* is a prevalent bacterial family in the ocean, especially in the nutrient-rich phycosphere. As typical copiotrophs [19,20], members of this family generally possess large genomes that enable the synthesis of most essential amino acids and vitamins, transport of diverse nutrients, and degradation of algae-derived macromolecules such as polysaccharides. However, our previous study [26] identified a unique *Alteromonadaceae* member, *Opacimonas immobilis* LMIT016^T^, which harbors the smallest recorded genome in the family and has lost genes essential for flagellar assembly and chemotaxis. Here, the comparative analyses reveal that strain LMIT016^T^ exhibits a pronounced reduction in genes associated with dynamic lifestyle switching, including a simplified c-di-GMP regulatory network. In contrast, gene clusters involved in extracellular polysaccharide biosynthesis, antimicrobial tolerance, phosphorus acquisition, and polysaccharide utilization are relatively retained. Together, these results suggest that strain LMIT016^T^ has undergone selective genomic reduction within the *Alteromonadaceae*, preferentially losing regulatory and motility-associated capacities while maintaining functional modules that support stable surface association and utilization of host-derived resources in the phycosphere.

Unlike most members of the *Alteromonadaceae*, strain LMIT016^T^ lacks flagellar and chemotaxis systems. These systems enable marine bacteria to sense environmental signal molecules and respond via motility, thereby providing a competitive advantage [51,52], particularly for heterotrophic bacteria that can sense and swim towards microalgal-derived organic compounds within the phycosphere [52,53]. The absence of motility could theoretically constrain the ability of strain LMIT016^T^ to compete for favorable niches in the dynamic marine environment. However, recent findings suggest that while chemotaxis facilitates initial bacteria–phytoplankton interactions, the competitive advantage of chemotaxis diminishes under high phytoplankton concentrations [1]. Thus, the loss of motility may confer less disadvantage during algal blooms. Intriguingly, LMIT016^T^ exhibits strong adhesion to diatom cells, suggesting an alternative strategy compensating for immotility. LMIT016^T^ notably harbors three complete Group I capsular polysaccharide (CPS) loci, along with full complements of lipopolysaccharide (LPS) biosynthesis genes, reflecting a substantial genomic investment in cell-surface polysaccharide production. In parallel, comparative genomic analyses indicate a pronounced reduction in c-di-GMP signaling components, a regulatory system widely implicated in controlling motile–sessile transitions in bacteria [37,38]. Collectively, these features are consistent with a shift toward an attachment lifestyle and suggest specialization for persistent association with diatom surfaces. The loss of motility together with enhanced adhesion may reflect a trade-off favoring stable surface attachment over active movement. However, the functional consequences of CPS and LPS biosynthesis capacity, as well as reduced c-di-GMP signaling, remain to be experimentally validated. Direct measurements of intracellular c-di-GMP levels and targeted phenotypic assays will be required in future studies to establish causal links between these genomic traits and attachment-related phenotypes.

Besides compensating for the loss of motility through stronger surface attachment, LMIT016^T^ also exhibited more stable growth in the phycosphere, suggesting a gene repertoire adapted for efficient energy conversion and nutrient acquisition. Diatoms continuously release polysaccharides such as laminarin and other dissolved carbohydrates into the phycosphere [54,55]. By retaining the “sharing” strategy of *Alteromonadaceae*, using cell-surface-associated or secreted enzymes to hydrolyze complex polymers into diffusible low-molecular-weight products [49], strain LMIT016^T^ is ideally positioned to benefit from its close physical association with the diatom host. Previous studies show that attached bacteria often exhibit greater diversity and activity of hydrolytic enzymes than free-living counterparts, enabling more efficient degradation of particulate or high-molecular-weight organic matter [56,57]. Consistent with this pattern, LMIT016^T^ combines both strong adhesion with the ability to utilize host polysaccharides, enhancing its capacity to exploit host polymers and persist in the phycosphere. Specifically, LMIT016^T^ encodes two PULs targeting laminarin and α-1,4-glucans, closely resembling validated modules in *Gramella forsetii* [58]. These PULs contain coordinated clusters of CAZymes, transporters, and central metabolic genes, supporting their role in algal polysaccharide degradation, consistent with the observed GHs induction and laminarin oligosaccharide depletion in the FACE analysis. Notably, laminarin and α-1,4-glucans are common polysaccharides produced by marine microalgae, especially diatoms and brown algae [59]. Therefore, the PUL repertoire of LMIT016^T^ likely represents an adaptation to generally available algal-derived carbohydrates, rather than host-specific dependence on a single algal host. In this context, stable association within the phycosphere is more likely driven by close physical proximity and continuous access to host-released polymers. In addition, the degradation of polysaccharides in the phycosphere may generate a microenvironment enriched with metabolic intermediates, potentially influencing interactions with nearby microbes [60]. Taken together, these features suggest a possible ecological strategy in which adhesion and targeted polysaccharide utilization underpin stable coexistence with algal hosts. The retention of streamlined yet functionally coherent polysaccharide utilization systems highlights a potential design principle for constructing stable algal–bacterial consortia, which are increasingly relevant for biotechnological applications such as algal cultivation, biomass conversion, and microbiome engineering. In addition, the specificity and efficiency of its polysaccharide-degrading enzymes toward widely distributed algal polymers may be of interest for enzyme discovery and antifouling strategies aimed at modulating early surface conditioning in marine environments [61,62].

At the genomic level, strain LMIT016^T^ exhibits a streamlined genome (~40% smaller than the average *Alteromonadaceae* genome), raising questions about its evolutionary origin. While LMIT016^T^ could represent an ancestral, pre-motile *Alteromonadaceae* state, its phylogenetic position within a derived rather than basal clade and the widespread presence of flagellar and chemotaxis systems in related lineages make this unlikely [26,63]. Instead, the targeted loss of motility, chemotaxis, and regulatory modules, coupled with the retention of adhesion, CAZymes, and specific stress-response functions, points to niche-driven streamlining. The phycosphere is a spatially stable and resource-enriched microenvironment, in which persistent association with phytoplankton hosts can reduce the selective advantage of motility and environmental sensing. Under such conditions, host dependence may act as a key ecological driver of genome reduction, favoring the retention of functions directly supporting surface association and host-derived resource utilization. Comparable patterns of selective genome reduction occur in diverse marine bacteria. For instance, the CHUG (Clade Hidden and Underappreciated Globally) lineage possesses one of the smallest genomes (~2.6 Mbp) among the Roseobacter group, having lost genes for vitamin B_12_ biosynthesis and certain algal metabolite utilization pathways, thereby optimizing a free-living lifestyle [64]. Moreover, LMIT016^T^ provides a valuable reference for exploring the genetic features involved in algal–bacterial interactions. Its specific pattern of gene loss and retention makes it a natural model for designing stable algal–bacterial consortia and for investigating the functional basis of phycosphere colonization, with broader implications for understanding micro-scale carbon processing in algal blooms and for the rational engineering of microbial consortia in algal-based biotechnological systems.

## 5. Conclusions

This study reveals that strain LMIT016^T^ follows a unique evolutionary pattern within the *Alteromonadaceae*, marked by a shift from a motile copiotrophic lifestyle to a non-motile, attachment-biased strategy driven by asymmetric genome reduction. By selectively eliminating energetically costly systems, including flagella, chemotaxis, and c-di-GMP signaling, while retaining and expanding functions essential for stable surface colonization, LMIT016^T^ facilitates stable attachment to diatom hosts. This adhesion strategy positions it for direct access to host-derived nutrients, compensating for lost motility and facilitating efficient exploitation of algal polysaccharides via specialized PULs targeting laminarin and α-1,4-glucans. Beyond advancing our understanding of genome streamlining in host-associated bacteria, these findings highlight a potential strategy for stable bacterial persistence within the phycosphere, with implications for nutrient exchange and micro-scale carbon cycling in marine ecosystems. Future work should extend these findings by examining interactions between LMIT016^T^ and multiple diatom species to assess the generality and host specificity of this attachment-biased lifestyle.

## Figures and Tables

**Table 1:** Genomic organization and functional annotation of genes within two polysaccharide utilization loci (PULs) in the genome of strain LMIT016^T^.

ID	Locus Tag	Length (aa)	Domains	Functions	EC Numbers
Laminarin PUL1	LMIT016_00395	454	GH1	Beta-glucosidase	3.2.1.21
	LMIT016_00396	420	GluP	Glucose/galactose transporter	
	LMIT016_00397	862	GH3	Beta-glucosidase	3.2.1.21
	LMIT016_00398	1012	TonB	Outer membrane receptor protein	
	LMIT016_00399	1599	GH16	Endo-β-1,3-glucanases	3.2.1.39
	LMIT016_00400	498	Gnd	Catalyzes the oxidative decarboxylation of 6-phosphogluconate to ribulose 5-phosphate	1.1.1.343
	LMIT016_00401	325	PfkB	Carbohydrate kinase	2.7.1.4
	LMIT016_00404	773	GH13	Alpha-amylase	3.2.1.1
	LMIT016_00405	152	MgsA	MGS-like	4.2.3.3
	LMIT016_00406	502	GlpK	Catalyzes the phosphorylation of glycerol to yield sn-glycerol-3-phosphate	2.7.1.30
	LMIT016_00407	257	GlpR	Transcriptional regulator	
	LMIT016_00408	513	GlpD	FAD-dependent glycerol-3-phosphate dehydrogenase	1.1.5.3
Alpha-1,4-glucan PUL2	LMIT016_02110	688	GH13	Alpha-amylase	3.2.1.68
	LMIT016_02111	725	GH13	Alpha-amylase	2.4.1.18
	LMIT016_02112	737	GH77	Alpha-1,4-glucanotransferase	2.4.1.25
	LMIT016_02113	336	GapA	Glyceraldehyde-3-phosphate dehydrogenase	1.2.1.12
	LMIT016_02114	270	YeaD	Glucose-6-phosphate 1-epimerase	5.1.3.15
	LMIT016_02115	219	Eda	2-keto-3-deoxy-6-phosphogluconate aldolase	4.1.2.14
	LMIT016_02116	330	Glk	Glucokinase	2.7.1.2
	LMIT016_02117	621	Edd	IlvD Edd family	4.2.1.12
	LMIT016_02118	232	Pgl	6-phosphogluconolactonase Glucosamine-6-phosphate isomerase deaminase	3.1.1.31
	LMIT016_02119	507	Zwf	Catalyzes the oxidation of glucose 6-phosphate to 6-phosphogluconolacton	1.1.1.363
	LMIT016_02120	285	HexR	Transcriptional regulator	

## Data Availability

The data presented in this study are openly available in NCBI GenBank at [https://www.ncbi.nlm.nih.gov/datasets/genome/GCF_048282545.1/, accessed on 6 March 2025], reference number [GCA_048282545.1].

## Supplementary Materials

The following supporting information can be downloaded at https://www.mdpi.com/article/10.3390/microorganisms14010139/s1, Table S1: Target genes and corresponding primer sequences used in this study. Figure S1: Fluorescence micrograph showing diatom-bacterial adhesion. Diatom cell walls were stained with Alcian Blue (blue). The DNA of diatoms and bacteria was stained with SYBR Green I (green). R1–R3 indicate three independent biological replicates. Scale bars: 20 μm (100× objective). Figure S2: Quantification of bacterial adhesion density and strain LMIT016T and two reference strains. Figure S3: Laminarin degradation by strain LMIT016T. Electrophoresis lanes (from left to right): Control (no bacterial inoculation), 0 h, 24 h, 48 h, 72 h and 96 h post-inoculation (bands shown).

## References

[1] Seymour J.R., Amin S.A., Raina J.-B., Stocker R. (2017). Zooming in on the phycosphere: The ecological interface for phytoplankton–bacteria relationships. Nat. Microbiol..

[2] Falkowski P.G. (1994). The role of phytoplankton photosynthesis in global biogeochemical cycles. Photosynth. Res..

[3] Longnecker K., Kido Soule M.C., Kujawinski E.B. (2015). Dissolved organic matter produced by *Thalassiosira pseudonana*. Mar. Chem..

[4] Bell W., Mitchell R. (1972). Chemotactic and Growth Responses of Marine Bacteria to Algal Extracellular Products. Biol. Bull..

[5] Buchan A., LeCleir G.R., Gulvik C.A., González J.M. (2014). Master recyclers: Features and functions of bacteria associated with phytoplankton blooms. Nat. Rev. Microbiol..

[6] Jiao N., Cai R., Zheng Q., Tang K., Liu J., Jiao F., Wallace D., Chen F., Li C., Amann R. (2018). Unveiling the enigma of refractory carbon in the ocean. Natl. Sci. Rev..

[7] Needham D.M., Fuhrman J.A. (2016). Pronounced daily succession of phytoplankton, archaea and bacteria following a spring bloom. Nat. Microbiol..

[8] Martin-Platero A.M., Cleary B., Kauffman K., Preheim S.P., McGillicuddy D.J., Alm E.J., Polz M.F. (2018). High resolution time series reveals cohesive but short-lived communities in coastal plankton. Nat. Commun..

[9] Croft M.T., Lawrence A.D., Raux-Deery E., Warren M.J., Smith A.G. (2005). Algae acquire vitamin B12 through a symbiotic relationship with bacteria. Nature.

[10] Amin S.A., Hmelo L.R., van Tol H.M., Durham B.P., Carlson L.T., Heal K.R., Morales R.L., Berthiaume C.T., Parker M.S., Djunaedi B. (2015). Interaction and signalling between a cosmopolitan phytoplankton and associated bacteria. Nature.

[11] Azam F., Malfatti F. (2007). Microbial structuring of marine ecosystems. Nat. Rev. Microbiol..

[12] Amin Shady A., Parker Micaela S., Armbrust E.V. (2012). Interactions between Diatoms and Bacteria. Microbiol. Mol. Biol. Rev..

[13] Yang X., Cai G., Cai R., Gu H., Chen Y., Xie J., Hu Z., Wang H. (2023). Redefinition of archetypal phytoplankton-associated bacteria taxa based on globally distributed dinoflagellates and diatoms. bioRxiv.

[14] Li D., He Y., Zheng Y., Zhang S., Zhang H., Lin L., Wang D. (2022). Metaproteomics reveals unique metabolic niches of dominant bacterial groups in response to rapid regime shifts during a mixed dinoflagellate bloom. Sci. Total Environ..

[15] Pedler B.E., Aluwihare L.I., Azam F. (2014). Single bacterial strain capable of significant contribution to carbon cycling in the surface ocean. Proc. Natl. Acad. Sci. USA.

[16] Baker B.J., Sheik C.S., Taylor C.A., Jain S., Bhasi A., Cavalcoli J.D., Dick G.J. (2013). Community transcriptomic assembly reveals microbes that contribute to deep-sea carbon and nitrogen cycling. ISME J..

[17] García-Martínez J., Acinas S.G., Massana R., Rodríguez-Valera F. (2002). Prevalence and microdiversity of *Alteromonas macleodii*-like microorganisms in different oceanic regions. Environ. Microbiol..

[18] Pfreundt U., Spungin D., Bonnet S., Berman-Frank I., Hess W.R. (2016). Global analysis of gene expression dynamics within the marine microbial community during the VAHINE mesocosm experiment in the southwest Pacific. Biogeosciences.

[19] Hou S., López-Pérez M., Pfreundt U., Belkin N., Stüber K., Huettel B., Reinhardt R., Berman-Frank I., Rodriguez-Valera F., Hess W.R. (2018). Benefit from decline: The primary transcriptome of *Alteromonas macleodii* str. Te101 during *Trichodesmium* demise. ISME J..

[20] Koch H., Dürwald A., Schweder T., Noriega-Ortega B., Vidal-Melgosa S., Hehemann J.-H., Dittmar T., Freese H.M., Becher D., Simon M. (2019). Biphasic cellular adaptations and ecological implications of *Alteromonas macleodii* degrading a mixture of algal polysaccharides. ISME J..

[21] Reintjes G., Fuchs B.M., Scharfe M., Wiltshire K.H., Amann R., Arnosti C. (2020). Short-term changes in polysaccharide utilization mechanisms of marine bacterioplankton during a spring phytoplankton bloom. Environ. Microbiol..

[22] Cusick K.D., Polson S.W., Duran G., Hill R.T. (2020). Multiple Megaplasmids Confer Extremely High Levels of Metal Tolerance in *Alteromonas* Strains. Appl. Environ. Microbiol..

[23] Clerc E.E., Raina J.-B., Keegstra J.M., Landry Z., Pontrelli S., Alcolombri U., Lambert B.S., Anelli V., Vincent F., Masdeu-Navarro M. (2023). Strong chemotaxis by marine bacteria towards polysaccharides is enhanced by the abundant organosulfur compound DMSP. Nat. Commun..

[24] McCutcheon J.P., Moran N.A. (2012). Extreme genome reduction in symbiotic bacteria. Nat. Rev. Microbiol..

[25] Giovannoni S.J., Cameron Thrash J., Temperton B. (2014). Implications of streamlining theory for microbial ecology. ISME J..

[26] Yang X., Lin X., Cai R., Wang H., Cai G. (2025). *Opacimonas immobilis* sp. nov., a novel non-flagellated *Alteromonadaceae* species with the smallest genome isolated from the phycosphere of the diatom *Actinocyclus curvatulus*. Arch. Microbiol..

[27] Pierella Karlusich J.J., Cosnier K., Zinger L., Henry N., Nef C., Bernard G., Scalco E., Dvorak E., Acinas S.G., Babin M. (2025). Patterns and drivers of diatom diversity and abundance in the global ocean. Nat. Commun..

[28] Guillard R.R.L., Smith W.L., Chanley M.H. (1975). Culture of Phytoplankton for Feeding Marine Invertebrates. Culture of Marine Invertebrate Animals: Proceedings —1st Conference on Culture of Marine Invertebrate Animals Greenport.

[29] Richard A.L., Farooq A. (1996). Abundant protein-containing particles in the sea. Aquat. Microb. Ecol..

[30] Palanisamy P., Crossia W.F., Prakash D., Antonyraj A.P.M. (2025). Nanoformulation of stavudine-loaded niosomes: Enhancing drug delivery, entrapment efficiency, and controlled release for improved antiretroviral therapy. J. Orthop. Rep..

[31] Zoccarato L., Sher D., Miki T., Segrè D., Grossart H.-P. (2022). A comparative whole-genome approach identifies bacterial traits for marine microbial interactions. Commun. Biol..

[32] Mann A.J., Hahnke R.L., Huang S., Werner J., Xing P., Barbeyron T., Huettel B., Stüber K., Reinhardt R., Harder J. (2013). The Genome of the Alga-Associated Marine Flavobacterium *Formosa agariphila* KMM 3901T Reveals a Broad Potential for Degradation of Algal Polysaccharides. Appl. Environ. Microbiol..

[33] Zech H., Thole S., Schreiber K., Kalhöfer D., Voget S., Brinkhoff T., Simon M., Schomburg D., Rabus R. (2009). Growth phase-dependent global protein and metabolite profiles of *Phaeobacter gallaeciensis* strain DSM 17395, a member of the marine Roseobacter-clade. Proteomics.

[34] Robb M., Hobbs J.K., Boraston A.B., Abbott D.W., Zandberg W.F. (2023). Separation and Visualization of Glycans by Fluorophore-Assisted Carbohydrate Electrophoresis. Carbohydrate-Protein Interactions: Methods and Protocols.

[35] Livak K.J., Schmittgen T.D. (2001). Analysis of Relative Gene Expression Data Using Real-Time Quantitative PCR and the 2^−ΔΔCT^ Method. Methods.

[36] Hussain S.A., Ghimouz R., Panda S.P., Panigrahy U.P., Marunganathan V., Shaik M.R., Deepak P., Thiyagarajulu N., Shaik B., Antonyraj A.P.M. (2025). Synergistic effects of copper oxide-stigmasterol nanoparticles: A novel therapeutic strategy for oral pathogen biofilms and oral cancer. Mater. Technol..

[37] Hengge R. (2009). Principles of c-di-GMP signalling in bacteria. Nat. Rev. Microbiol..

[38] Khan F., Jeong G.-J., Tabassum N., Kim Y.-M. (2023). Functional diversity of c-di-GMP receptors in prokaryotic and eukaryotic systems. Cell Commun. Signal..

[39] Whitfield C., Trent M.S. (2014). Biosynthesis and Export of Bacterial Lipopolysaccharides*. Annu. Rev. Biochem..

[40] Hinsa S.M., Espinosa-Urgel M., Ramos J.L., O’Toole G.A. (2003). Transition from reversible to irreversible attachment during biofilm formation by *Pseudomonas fluorescens* WCS365 requires an ABC transporter and a large secreted protein. Mol. Microbiol..

[41] Whitfield C., Wear S.S., Sande C. (2020). Assembly of Bacterial Capsular Polysaccharides and Exopolysaccharides. Annu. Rev. Microbiol..

[42] Smith B.L., Fernando S., King M.D. (2024). *Escherichia coli* resistance mechanism AcrAB-TolC efflux pump interactions with commonly used antibiotics: A molecular dynamics study. Sci. Rep..

[43] Zhu Y., Dou Q., Du L., Wang Y. (2023). QseB/QseC: A two-component system globally regulating bacterial behaviors. Trends Microbiol..

[44] Mettert E.L., Kiley P.J. (2015). How Is Fe-S Cluster Formation Regulated?. Annu. Rev. Microbiol..

[45] Saavedra D.E.M., González J.M., Klaushofer K., Breyer E., Afjehi-Sadat L., Bulgheresi S., Liao L., Dong X., Patrick W.M., Baltar F. (2025). Multifunctionally diverse alkaline phosphatases of *Alteromonas* drive the phosphorus cycle in the ocean. Nat. Commun..

[46] Olofsson M., Ferrer-González F.X., Uchimiya M., Schreier J.E., Holderman N.R., Smith C.B., Edison A.S., Moran M.A. (2022). Growth-stage-related shifts in diatom endometabolome composition set the stage for bacterial heterotrophy. ISME Commun..

[47] Percival E.G.V., Ross A.G. (1951). 156. The constitution of laminarin. Part II. The soluble laminarin of *Laminaria digitata*. J. Chem. Soc..

[48] Michel G., Tonon T., Scornet D., Cock J.M., Kloareg B. (2010). Central and storage carbon metabolism of the brown alga *Ectocarpus siliculosus*: Insights into the origin and evolution of storage carbohydrates in Eukaryotes. New Phytol..

[49] Reintjes G., Arnosti C., Fuchs B.M., Amann R. (2017). An alternative polysaccharide uptake mechanism of marine bacteria. ISME J..

[50] Ramanan R., Kim B.-H., Cho D.-H., Oh H.-M., Kim H.-S. (2016). Algae–bacteria interactions: Evolution, ecology and emerging applications. Biotechnol. Adv..

[51] Raina J.-B., Fernandez V., Lambert B., Stocker R., Seymour J.R. (2019). The role of microbial motility and chemotaxis in symbiosis. Nat. Rev. Microbiol..

[52] Stocker R., Seymour J.R. (2012). Ecology and Physics of Bacterial Chemotaxis in the Ocean. Microbiol. Mol. Biol. Rev..

[53] Raina J.-B., Giardina M., Brumley D.R., Clode P.L., Pernice M., Guagliardo P., Bougoure J., Mendis H., Smriga S., Sonnenschein E.C. (2023). Chemotaxis increases metabolic exchanges between marine picophytoplankton and heterotrophic bacteria. Nat. Microbiol..

[54] Becker S., Tebben J., Coffinet S., Wiltshire K., Iversen M.H., Harder T., Hinrichs K.-U., Hehemann J.-H. (2020). Laminarin is a major molecule in the marine carbon cycle. Proc. Natl. Acad. Sci. USA.

[55] Gügi B., Le Costaouec T., Burel C., Lerouge P., Helbert W., Bardor M. (2015). Diatom-Specific Oligosaccharide and Polysaccharide Structures Help to Unravel Biosynthetic Capabilities in Diatoms. Mar. Drugs.

[56] Hoppe H.G., Marshall K.C. (1984). Attachment of Bacteria: Advantage or Disadvantage for Survival in the Aquatic Environment. Microbial Adhesion and Aggregation.

[57] Leu Andy O., Eppley John M., Burger A., DeLong Edward F. (2022). Diverse Genomic Traits Differentiate Sinking-Particle-Associated versus Free-Living Microbes throughout the Oligotrophic Open Ocean Water Column. mBio.

[58] Kabisch A., Otto A., König S., Becher D., Albrecht D., Schüler M., Teeling H., Amann R.I., Schweder T. (2014). Functional characterization of polysaccharide utilization loci in the marine Bacteroidetes ‘Gramella forsetii’ KT0803. ISME J..

[59] Beidler I., Steinke N., Schulze T., Sidhu C., Bartosik D., Zühlke M.-K., Martin L.T., Krull J., Dutschei T., Ferrero-Bordera B. (2024). Alpha-glucans from bacterial necromass indicate an intra-population loop within the marine carbon cycle. Nat. Commun..

[60] Mühlenbruch M., Grossart H.-P., Eigemann F., Voss M. (2018). Mini-review: Phytoplankton-derived polysaccharides in the marine environment and their interactions with heterotrophic bacteria. Environ. Microbiol..

[61] Zanaroli G., Negroni A., Calisti C., Ruzzi M., Fava F. (2011). Selection of commercial hydrolytic enzymes with potential antifouling activity in marine environments. Enzym. Microb. Technol..

[62] Kristensen J.B., Meyer R.L., Laursen B.S., Shipovskov S., Besenbacher F., Poulsen C.H. (2008). Antifouling enzymes and the biochemistry of marine settlement. Biotechnol. Adv..

[63] Ivanova E.P., Mikhailov V.V. (2001). A New Family, *Alteromonadaceae* fam. nov., Including Marine Proteobacteria of the Genera *Alteromonas*, *Pseudoalteromonas*, *Idiomarina*, and *Colwellia*. Microbiology.

[64] Feng X., Chu X., Qian Y., Henson M.W., Lanclos V.C., Qin F., Barnes S., Zhao Y., Thrash J.C., Luo H. (2021). Mechanisms driving genome reduction of a novel Roseobacter lineage. ISME J..

