# Supplementary material for: Polysaccharide Utilization and Adhesion Enable the Genome-Streamlined Opacimonas immobilis to Adapt to the Diatom Phycosphere
Source: Microorganisms. 2026 Jan 8;14(1):139. doi: 10.3390/microorganisms14010139 (PMC12844025; doi:10.3390/microorganisms14010139)
Supplement: Supplementary file 1 [file microorganisms-14-00139-s001.zip › microorganisms-4049737-supplementary.pdf]

Table S1. Target genes and corresponding primer sequences used in this study.

Gene	Direction	Sequence (5'–3')
GH1	Forward	AAGGCGACCGAGAATGGATG
	Reverse	TGGCGGCAGTGAATATCGTT
GH3	Forward	CACGCAGTCAATTGGTGTGG
	Reverse	ACTCCAGCATAAGCACCACC
GH16	Forward	TTTGTGCCTGAAGGTAGCCC
	Reverse	AAGTACGAGCTTCCGAACCG
16S rRNA (Internal control)	Forward	ACGGGTGAGTAATGCTTGGG
	Reverse	GAGCCTAAGCCCACTTTGGT

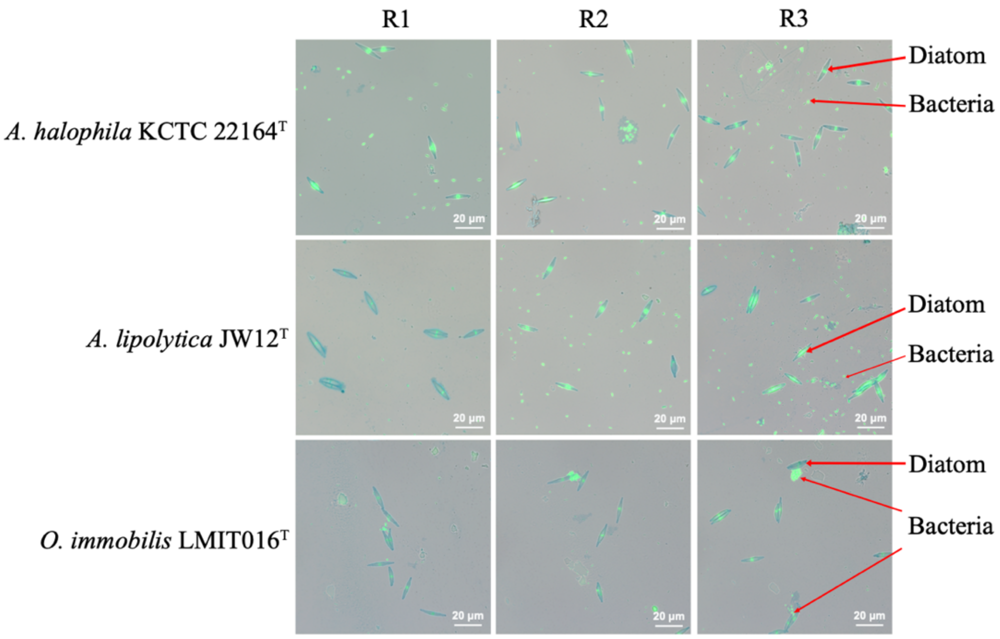

Figure S1. Fluorescence micrograph showing diatom-bacterial adhesion. Diatom cell walls were stained with Alcian Blue (blue). The DNA of diatoms and bacteria was stained with SYBR Green I (green). R1–R3 indicate three independent biological replicates. Scale bars: 20 µm (100× objective).

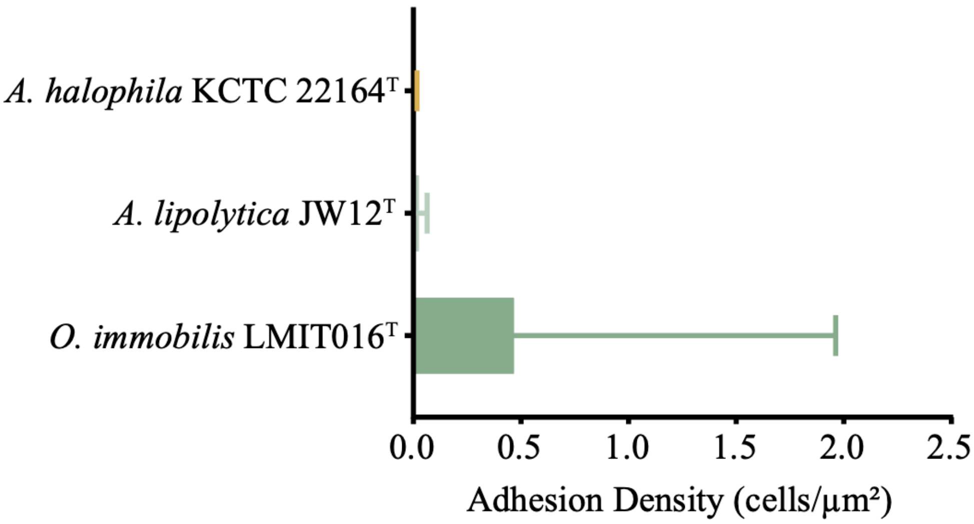

Figure S2. Quantification of bacterial adhesion density and strain LMIT016^T and two reference strains.

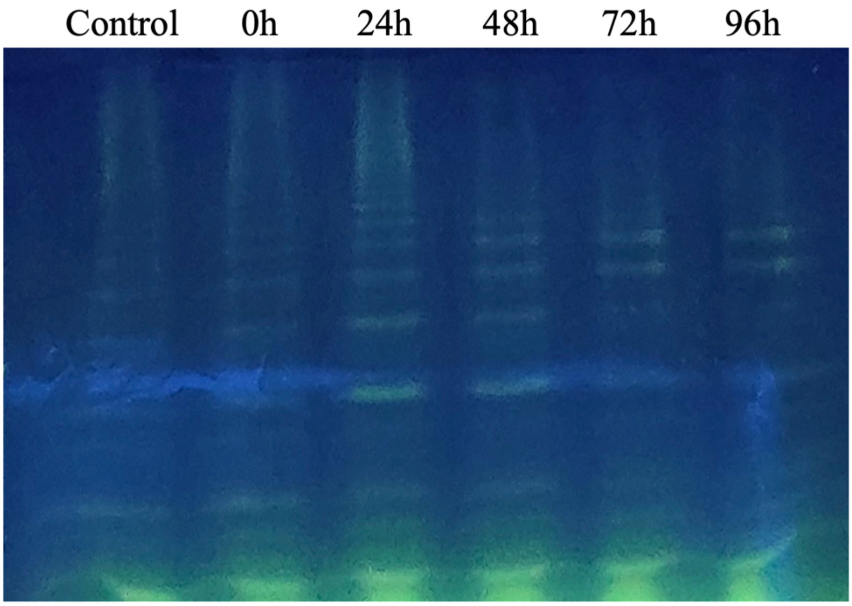

Figure S3. Laminarin degradation by strain LMIT016^{T}. Lane 1: Control (no bacterial inoculation); Lane 2-6: Degradation profiles at 0 h, 24 h, 48 h, 72 h and 96 h, respectively (electrophoretic bands shown).
